# Medical and Physical Intelligence

**Published:** 1805-11-01

**Authors:** 


### MEDICAL AND PHYSICAL INTELLIGENCE.

[ FOREIGN AND DOMESTIC. ]

### *Effects of the Alkalies on Poisons.*

THE excellence of alkaline salts, as antidotes to the venom of serpents, and the acidity of the poisons secreted by those animals, is evinced by the following information, written January 7, 1805, by Dr. John Brickell, of Savannah, to Dr. Daniel M. Hitchcock, of New York:—"You have probably seen my publication, of a successful mode of cure on a person bit by a large mockison snake. He was in dreadful torture, and swelled ready to burst. I gave him plenty of ley of ashes with water to drink, and kept the bite which was on his foot constantly wet, by applying a solution of ley made caustic by quick lime.

"I was

"I was led to this by a chemical examination of the poison of crotalus horridus, which showed an acid to be one of its constituent parts.

"The mechanical structure of the poison-fangs and bags is alike in each of the above serpents.

"On putting a slip of litmus paper into the mouth of a rattle-snake five feet long, and disabled by blows of a stick applied to the back, and then pressing the head, by placing on it the foot, the poison squirted out to the distance of some feet.

"Some of the poison struck above the eye of the gentleman who performed the operation for me; he wiped it off with his pocket handkerchief and no harm ensued; some struck on his white cotton waistcoat, and tinged it of a light yellowish colour, inclining to brown.

"The part of the litmus paper, which I made of sky blue, had its blue *entirely discharged*, and became white. A light band of red was to be seen between the part of the paper touched and untouched by the poison. Dipping it, when dry, in a solution of fixed alkali (ley) restored the former blue colour.

"We have here, 1st. the crotalus horridus of Linnæus. 2d. the crotalus miliaris of Linnæus; and four species, new to me. One is nearly black, with a chain of white lined rhombi on the back: this I have named *crotalus rhombiferus;* this measured six feet two inches in length, and above 11 ¼ inches round.

"Another has a yellow skin, with zigzag brown belts, six feet long: this I have named *crotalus zetazona.* The 3d and 4th are twelve to fifteen inches long, venomous as the others; one is black, with paler bands down its sides; the other is yellowish, with a chain of rhomboid dark lines on the back, and a brown line round the head above the eyes."

---

### *Fatal Sting of a Wasp.*

A Correspondent informs us, that on Monday, September 2, Mr. Sperrey, of Diseworth, in the county of Leicester, was stung in a vein upon the back of his hand, which put a period to his existence on the following day. This fatal occurrence induces our Correspondent to submit two questions to the consideration of our readers, *viz.*

1. What is the immediate physical cause of death in such a case?
2. What are the most effectual means of preventing the ill effects of poison inserted by the sting of a wasp?

---

Mr. Van Mons has found broth keep for many years by means of a few grains of mercury in the state of oxide and citrate. Nitrate of silver has long been considered as the most powerful of antiseptics, and those of gold and mercury are equally so. Oxygenated muriate of pot-ash retarded the putrefaction of strong soup several several days, and ultimately put a stop to it at a certain point. Very dilute nitric acid and oxygenated muriatic acid preserved soup for several months.

---

Dr. FAUST, in conjunction with Dr. HUNOLD, of Cassel, will speedily publish a work, in which they will demonstrate that, excepting the lancet employed in vaccination, all the instruments of surgery ought to be dipped into oil at the moment when they are going to be used; by which method the pain of the subject operated upon will always be diminished. In the same work it is recommended to make all instruments of a blood-heat a little before the operation. These two precautions have already been practised in certain cases, and with certain instruments.

---

Dr. JAMES SIMS has transmitted a letter to the Editors, of which the following is an extract.

"Having read a paper to the Medical Society of London, in which I give my opinion that the Cow-pock is the Small-pox of mankind, translated to the cow by means of a species of inoculation, and afterwards reinserted into the human body in a mitigated form; which paper has been inserted in the sixth volume of their Memoirs: but as a part of that paper was not inserted, owing to an inattention of my own, I now take the liberty of addressing myself to you, for the purpose of correcting the lapse. The part of my opinion which was not stated is, that the spurious Cow-pock, as it is called, is nothing more than the swine or chicken-pock of the human body, transferred in a similar manner to and from that animal. This supposition easily accounts for a number of the failures recorded of vaccination, which have arisen from not accurately distinguishing one kind of pock from the other; and it so naturally fell in with my theory, that I am now surprised how, in correcting the press, I did not perceive the omission."

*Finsbury Square, Oct. 5,* 1805

---

Mr. BIRCH and Mr. WALKER, in a note to the Editors, deny that they pronounced the eruption on the child in Midford Place to be small-pox, as stated in our last Journal.

---

*Dr. Batty requests his private Correspondents will address their letters to him in future, at No. 27, Charlotte-Street, Portland Place, where he intends to reside after the 10th inst. Communications to him for the Medical and Physical Journal, are to be sent as usual to the care of Mr. Phillips.*

